# Unconstrained Vital Sign Monitoring System Using an Aortic Pulse Wave Sensor

**DOI:** 10.1038/s41598-019-53808-9

**Published:** 2019-11-25

**Authors:** Naoki Hagiyama, Harutoyo Hirano, Akihisa Mito, Zu Soh, Etsunori Fujita, Yumi Ogura, Shigehiko Kaneko, Ryuji Nakamura, Noboru Saeki, Masashi Kawamoto, Masao Yoshizumi, Toshio Tsuji

**Affiliations:** 1 Department of System Cybernetics, Graduate School of Engineering, Hiroshima University, 1-4-1 Kagamiyama, Higashi-Hiroshima, Hiroshima, 739-8527 Japan; 2 Academic Institute, College of Engineering, Shizuoka University, 3-5-1, Johoku, Naka-ku, Hamamatsu, Shizuoka 432-8561 Japan; 3 Department of System Cybernetics, Faculty of Engineering, Hiroshima University, 1-4-1 Kagamiyama, Higashi-Hiroshima, Hiroshima, 739-8527 Japan; 4 Delta Kogyo Co., Ltd., 1-14 Shinchi, Fuchu-cho, Aki-Gun, Hiroshima, 735-8501 Japan; 5 Major in Mechanical Engineering, School of Creative Science and Engineering, Center for Science and Engineering, Waseda University, 60-105, 3-4-1 Okubo, Shinjuku-ku, Tokyo 169-8555 Japan; 6 Department of Anesthesiology and Critical Care, Graduate School of Biomedical and Health Sciences, Hiroshima University, 1-2-3 Kasumi, Minami-ku, Hiroshima, Hiroshima, 734-8553 Japan; 7 Department of Cardiovascular Physiology and Medicine, Graduate School of Biomedical and Health Sciences, Hiroshima University, 1-2-3 Kasumi, Minami-ku, Hiroshima, Hiroshima, 734-8553 Japan

**Keywords:** Signal processing, Signal processing, Signal processing, Disease prevention, Disease prevention

## Abstract

This paper proposes a novel unconstrained monitoring system that measures heart and respiratory rates and evaluates autonomic nervous activity based on heart rate variability. The proposed system measures the aortic pulse waves (APWs) of a patient via an APW sensor that comprises a single microphone integrated into a mattress. Vital signs (i.e., heart rate, respiratory rate) and autonomic nervous activity were analyzed using the measured APWs. In an experiment with supine and seated participants, vital signs calculated by the proposed system were compared with vital signs measured with commercial devices, and we obtained the correlations of *r* > 0.8 for the heart rates, *r* > 0.7 for the respiratory rates, and *r* > 0.8 for the heart rate variability indices. These results indicate that the proposed system can produce accurate vital sign measurements. In addition, we performed the experiment of image stimulus presentation and explored the relationships between the self-reported psychological states evoked by the stimulus and the measured vital signs. The results indicated that vital signs reflect psychological states. In conclusion, the proposed system demonstrated its ability to monitor health conditions by actions as simple as sitting or lying on the APW sensor.

## Introduction

In 2013, 25% of the population of Japan was aged 65 or older, and it is expected to exceed 30% in 2025^1^. This rapid increase in the aging population increases the need for the daily monitoring of the health conditions of elderly and bedridden patients. Numerous researchers reported that monitoring vital signs such as heart rate, heart rate variability (HRV) indices^2^, and respiratory rate during daily life is effective for the early detection of disease^3–5^. This indicates the usefulness of home care systems that monitor heart rate, respiratory rate, and HRV indices.

To this end, wearable devices were developed to facilitate the measurement of vital signs such as heart rate and respiratory rate, and to monitor acute deterioration^6–8^. However, most of these devices require the use of sensors directly attached to the body, which induces stress during long-term measurement.

Therefore, unconstrained health monitoring methods have been proposed to solve the above issue^9–17^. Systems that embedded capacitive electrodes^9–12^ and microwave radar^13,14^ in locations such as chairs and beds enabled continuous heart monitoring in daily life. Pulse waves have been extracted with high accuracy from the change in the brightness of images for human skin owing to advances in photonics technology^17^. Some studies also reported that HRV indices can be calculated using the cardiovascular blood volume pulse extracted from recorded video images of human skin^15,16^. These previous systems could measure vital signs with minimal burden on users. However, the highest sampling rate of the video analysis system (200 Hz)^14–16^ was below the recommended sampling rate (over 250 Hz) for accurately calculating HRV indices.

We thus focused on an aortic pulse wave (APW) sensor with 1000 Hz sampling frequency that was previously fabricated by our research group^18–20^. The APW sensor is integrated into a mattress constructed using 3D-NET^19,21^, a fabric that can simulate the mechanical characteristics of human muscle. As a result, the mattress can appropriately distribute the body weight, and thus, prevent decubitus. The sensor includes a capacitor-type microphone and an intrinsic oscillator, such that aortic pulse waves resonated by the oscillator are measured by the microphone. Measured aortic pulse waves are considered to contain cardiac, vascular, and respiratory information^20^; however, their extraction algorithms have not been established.

Against this background, this paper presents a novel algorithm for extracting the indices related to HRV and respiratory rate; additionally, artefacts caused by body movements are removed using mechanical and digital filters. To verify the accuracy of the proposed algorithm with both supine and seated patients, we compared the extracted indices against measurements obtained using commercially available instruments. Further, we performed image simulation experiments to test whether the extracted indices could be used for evaluating psychological conditions.

## Materials and Methods

**Figure 1:**
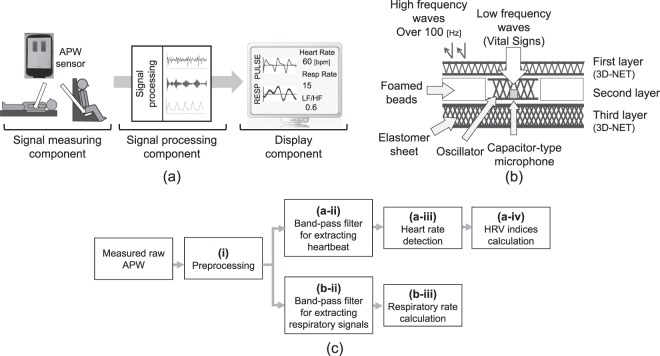
The proposed system: (**a**) overview of the proposed system, (**b**) structure of the APW sensor, and (**c**) algorithm for extracting vital signs from the measured APWs.

Figure 1(a) shows an overview of the proposed system, consisting of a measurement component that measures APW, a signal processing component that extracts vital signs from measured APWs, and a display component that displays extracted results. Study participants are in a supine or sitting position while their APWs are measured with the APW sensor. The details of the proposed system are described below.

### APW sensor

Figure 1(b) shows an overview of the APW sensor used in the measurement component. The APW sensor includes a capacitor-type microphone sensor and consists of three layers. The first and third layers are constructed using 3D-NET, a three-dimensional solid knitted fabric^19,21^. The thicknesses of these layers are 10 and 7 mm, respectively. 3D-NET has mechanical characteristics similar to those of human muscle. It deforms significantly when local pressure is applied, and resists surface deformation when widespread pressure is applied; thus, it reduces the burden on the peripheral circulatory system. The pile density of the third layer is higher than that of the first layer, which allows APWs to be measured without causing discomfort to the patient. The second layer consists of a seat frame of polypropylene bead foam, an intrinsic oscillator with a centre frequency of approximately 20 Hz, and a capacitor-type microphone sensor. The surface of the second layer is covered with a sheet of polyester elastomeric film. The intrinsic oscillator is composed of 3D-NET monofilaments, and the bead foam sheet functions as a resonance box. Moreover, the bead foam sheet acts as a low-pass filter whose cutoff frequency can be expressed as

1 $${f}_{0}=\frac{1}{2\pi }\sqrt{\frac{k}{m}},$$

where the spring constant *k* and mass *m* of the bead foam sheet are 1279 kN/m and 3.4 g, respectively. The calculated cutoff frequency of the bead foam sheet is 96 Hz^19^. The sheet thus attenuates high-frequency (about 100 Hz or greater) disturbances. The signals originating from cardiovascular oscillation are propagated to the second layer via clothes, skin, muscle, and fat. The propagated signals, with a frequency of approximately 20 Hz, are amplified in the second layer by the intrinsic oscillator. The amplified signals are measured with the microphone sensor in the second layer. 3D-NET allows the measurement of APWs with high sensitivity.

### Proposed algorithm for extracting vital signs

This section describes the signal processing component. Figure 1(c) shows an overview of the proposed algorithm for extracting vital signs from the measured APWs. The processing methods are described below. In the present study, the second-order Butterworth bandpass filter is applied to the APW signal.

#### Preprocessing

To obtain vascular information, the measured APWs are filtered through a band-pass filter (low cutoff: 10 Hz; high cutoff: 30 Hz). To extract the cardiovascular information from the filtered waves, first-order differentiation and full-wave rectification are applied to the filtered APWs.

#### Heart rate

The method for extracting the heart rate (HR_APW_) is described below. To obtain the pulse wave PULSE_APW_, a bandpass filter with cutoff frequencies of 0.5 Hz and 3.0 Hz is applied to the filtered full-wave rectification waveforms described in Fig. 1(c),a-(ii). PULSE_APW_ has a peak corresponding to the heartbeat. The peak time, *t*_*i*_, of PULSE_APW_ is detected using the MATLAB function “findpeaks^22^”. Here, the minimum time interval between the adjacent peaks were set to be 400 ms for denoising. To eliminate peak time outliers, the Smirnov–Grubbs test^23^ is applied to the amplitudes of PULSE_APW_ at the obtained peak times. This is necessary because the amplitudes of PULSE_APW_ that overlap disturbances, such as those caused by body movement, may be substantially higher than those of normal pulse wave PULSE_APW_. The significance level of the test is set at *p* < 0.05. After the disturbances are eliminated, the peak-to-peak timings of PULSE_APW_ are calculated as the time interval (RRI_APW_ = *t*_*i*+1_ − *t*_*i*_) corresponding to the R-R interval of the electrocardiogram. Next, RRI_APW_ is tested with the Smirnov–Grubbs test to detect outliers caused by the algorithm error. RRI_APW_ are resampled at a frequency of 4 Hz^24^ by applying the three-dimensional spline interpolation method. HR_APW_ is expressed as

2 $${{\rm{HR}}}_{{\rm{APW}}}=\frac{60}{{{\rm{RRI}}}_{{\rm{APW}}}}.$$

#### Heart rate variability

The autonomic nerve consists of the sympathetic nerve and the parasympathetic nerve. The sympathetic nerve, which becomes activated during times of pain or stress, increases the heartbeat. The parasympathetic nerve, which becomes activated during times of relaxation or sleep, decreases the heartbeat. HRV indices are useful for the analysis of autonomic nervous activity. Previous studies reported that low-frequency (LF) power (0.04–0.15 Hz) reflects both parasympathetic and sympathetic nervous activity, high-frequency (HF) power (0.15–0.40 Hz) reflects parasympathetic nervous activity, and LF:HF ratio (LF/HF) reflects sympathetic nervous activity^2^. We thus calculated the power spectral density (PSD) of RRI_APW_. The periodogram method^25^, a common method for calculating PSD, was applied in this study. A previous study reported that the PSD analysis on the window size of the RR interval should be 50 s or greater to ensure reliability^26^. Therefore, we applied the Hamming window with the window size of 60 and the overlap time of 50 s. Based on the obtained PSD of RRI_APW_, low-frequency power and high-frequency power are determined and denoted as LF_APW_ and HF_APW_, respectively. LF_APW_ and HF_APW_ are then standardized by the following Eqs (3) and (4)^27^. In addition, LF:HF ratio is calculated by Eq. (5).

3 $${{\rm{LFnu}}}_{{\rm{APW}}}=\frac{{{\rm{LF}}}_{{\rm{APW}}}}{{{\rm{LF}}}_{{\rm{APW}}}+{{\rm{HF}}}_{{\rm{APW}}}},$$

4 $${{\rm{HFnu}}}_{{\rm{APW}}}=\frac{{{\rm{HF}}}_{{\rm{APW}}}}{{{\rm{LF}}}_{{\rm{APW}}}+{{\rm{HF}}}_{{\rm{APW}}}},$$

5 $${\rm{LF}}/{{\rm{HF}}}_{{\rm{APW}}}=\frac{{{\rm{LFnu}}}_{{\rm{APW}}}}{{{\rm{HFnu}}}_{{\rm{APW}}}}.$$

#### Respiratory rate

The method for extracting the respiratory rate (RR_APW_) is described below. To obtain RESP_APW_, a bandpass filter with cutoff frequencies of 0.15 Hz and 0.40 Hz is applied to the full-wave rectification waveforms. To extract the respiratory rate, the peak time, *t*_*i*_, of RESP_APW_ is detected using the MATLAB function “findpeaks^22^”. Here, the minimum time interval between the adjacent peaks were set to be 2500 ms for denoising. The number of peaks in RESP_APW_ is calculated as the respiratory rate RR_APW_ breaths/min.

Finally, the results obtained in the signal processing component, including HR_APW_, LFnu_APW_, HFnu_APW_, LF/HF_APW_, and RR_APW_, are displayed by the display component.

### Experimental configurations

To test the accuracy of the proposed algorithm, we simultaneously measured the HRV indices and respiratory rate using the proposed system and the commercially available sensors, and we compared their results. We then performed image stimulation experiments to examine the feasibility of using the extracted indices for evaluating the affect of participants. In accordance with the Declaration of Helsinki, informed consent was obtained from all study participants before the experiments were performed. The experimental protocol was approved by the Hiroshima University Ethics Committee (Registration Number: E-17-2).

#### Experiment to verify the measurement accuracy of the proposed system

**Figure 2:**
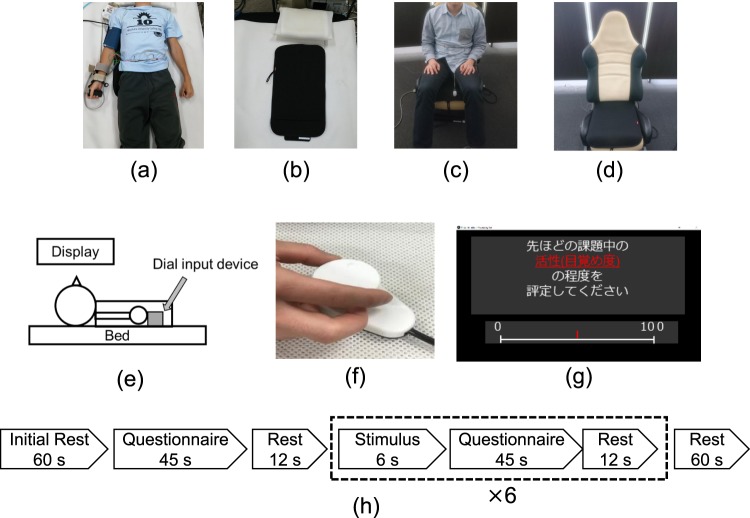
Environment used for APW measurement experiments during rest: (**a**) a participant in the supine position, (**b**) the APW sensor attached to a bed, (**c**) a participant in the sitting position, and (**d**) the APW sensor attached to a seat; Environment used for experiment on evaluation of affect: (**e**) a participant during measurement, (**f**) dial input device, (**g**) example of questionnaire in Japanese (the instruction reads, “Please rate your level of arousal during the previous task”), and (**h**) experimental protocol.

Figure 2(a–d) show the environment used for verifying the accuracy of the proposed system.

Twenty-nine healthy male participants (mean age ± standard deviation (SD): 22.9 ± 0.8 years) were recruited for the experiment. Sixteen participants (mean age ± SD: 22.8 ± 0.8 years) were instructed to assume a supine position; the remaining 13 participants (mean age ± SD: 23.0 ± 0.9 years) were instructed to assume a sitting position.

APW sensors measured APWs from the backs and hips of the supine and seated participants, respectively. Simultaneously, respiratory (RESP) waves were measured from the abdomen of the participant with a strain gauge^28^ employing a multichannel telemeter system (WEB-7000, NIHON KOHDEN), and electrocardiograms (ECGs) were recorded using a commercial physiological monitor (BP-608 Evolution II CS, OMRON COLIN Co.). All data were recorded for 180 s at a sampling frequency of 1000 Hz and stored on a PC using a data storage device (USB-6215, National Instruments).

To extract HR_APW_, LFnu_APW_, HFnu_APW_, and LF/HF_APW_, the measured APWs were processed using the proposed algorithm described in the heart rate and heart rate variability sections. In addition, the mean value of HR_APW_ for 60 s (Mean HR_APW_) was calculated every 10 s.

To obtain the heart rate (HR), R-peaks were detected in the ECGs. The HRs were resampled at a frequency of 4 Hz by applying a three-dimensional spline interpolation method. To obtain reference HRV indices, LFnu, HFnu, and LF/HF were calculated. In addition, the mean value of HR for 60 s (mean HR) was calculated every 10 s. To obtain the reference respiratory rate, the number of RESP peaks was calculated, and RR was extracted using the method described in the respiratory rate section.

To evaluate the accuracy of the extracted indices, the correlations between the indices extracted from APWs and those measured by the commercial instruments described in the previous paragraph were calculated. In addition, to evaluate the errors in the extracted HR, mean HR, LFnu, HFnu, LF/HF, and RR, a Bland–Altman analysis^29^ was conducted.

#### Experiment to verify the evaluability of affect in the proposed system

To confirm that the vital signs extracted using the proposed system could accurately reflect affect, APWs were measured when image stimuli were displayed. Figure 2(e) shows the environment used during the experiment to verify the accuracy of the proposed system.

Sixteen healthy male participants (mean age ± SD: 22.3 ± 1.0 years) were recruited for the experiment. All participants were instructed to be seated and watch a video display while wearing noise-cancelling headphones to reduce the impact of auditory stimuli. APWs were measured from the back of the participant using an APW sensor. The measured APWs were stored on a PC using a data storage device (USB-6215, National Instruments) at a sampling frequency of 1000 Hz.

The participants were asked to report the subjective sensation of affect based on the visual analogue scale (VAS)^30^, which ranks the level of affect in 101 increments from 0 to 100 (0 = no affect, 100 = maximum affect). Answers to the questionnaire were submitted using a dial input device, which is shown in Fig. 2(f). Figure 2(g) shows an example of the questionnaire. The questionnaire items, based on Russell’s circumplex model of affect^31^, were aroused, excited, pleased, relaxed, sleepy, bored, displeased, and irritated. Figure 2(h) shows the experiment protocol.

A task consisted of 6 s of image stimulation, 45 s of self-reported affect evaluation, and 12 s of rest. Six tasks with different image stimuli were sequentially performed. The experiment protocol was defined according to the following sequence: 1 min of initial rest, 45 s of self-reported affect evaluation, 12 s of rest, 6 task performances, and 60 s of rest. The image stimuli were selected from the International Affective Picture System^32^. For each participant, three positive images were randomly selected from among image numbers 1440, 1460, 1463, 1540, 1590, 1610, 1710, 1750, and 1920, and three negative images were randomly selected from among image numbers 3000, 3051, 3060, 3068, 3069, 3071, 3100, 3101, and 3266^33^. The display showed a white cross during the resting states. HR_APW_ and RESP_APW_ were extracted from the measured APWs using the proposed algorithm. The extracted HR_APW_ and RESP_APW_ were standardized to a normal distribution with mean 0 and standard deviation (SD) 1 for the entire experiment. The mean and SD values of the standardized HR_APW_ and the standardized RESP_APW_ recorded during the period of rest lasting from 9 s to 3 s before the start of the image stimulus were compared against those from the image stimulus interval. The differences were considered significant at *p* < 0.05.

The self-reported affect values were standardized for each participant. Principal component analysis (PCA) was applied to the standardized self-reported affect values to identify components whose cumulative contribution rate exceeded 80%. The correlation coefficients between the extracted vital signs and the affect values (standardized self-reported affect value and the extracted principal component scores) were calculated.

## Results

### Accuracy of vital signs extracted by the proposed system

**Figure 3:**
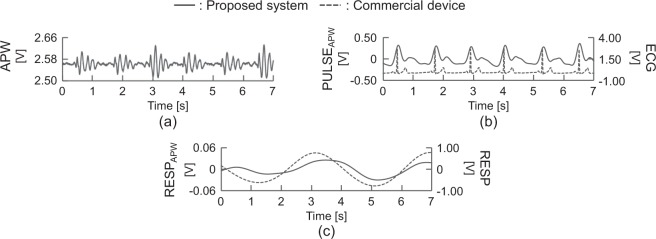
Measured waveforms of (**a**) APWs, (**b**) PULSE_APW_ and ECG, and (**c**) RESP_APW_ and RESP from participant A in the sitting position.

Figure 3 shows the measured waveforms for participant A. The periodic sharp peaks were confirmed at almost every 1 s from the measured APWs corresponding to the heartbeat. The timing of the peaks of PULSE_APW_ was almost equal to that of the ECG. The shape of RESP_APW_ was also equal to the RESP waves.

Table 1 shows the relationships between the extracted indices (HR, RR, LFnu, HFnu, and LF/HF) from the proposed system and those from the commercial instruments. The correlation coefficients between the HR, mean HR, HRV indices (LFnu, HFnu, and LF/HF), and RR extracted using the proposed system and those measured by the commercial instruments were greater than 0.7 (*p* < 0.001).

**Table 1:** Correlation coefficients of extracted indices from the proposed system and the commercial instruments.

	HR	Mean HR	LFnu	HFnu	LF/HF	RR
In supine position	0.96***	1.00***	0.90***	0.90***	0.93***	0.80***
In sitting position	0.88***	0.99***	0.85***	0.85***	0.81***	0.70***

***p < 0.001.

**Figure 4:**
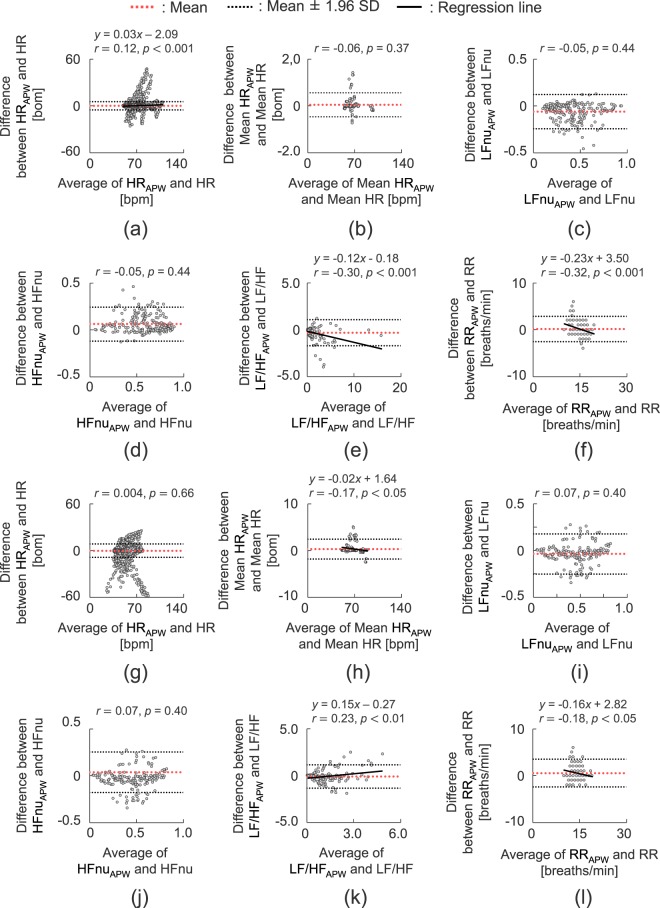
Bland–Altman plots of (**a**) HR, (**b**) mean HR, (**c**) LFnu, (**d**) HFnu, (**e**) LF/HF, and (**f**) RR in the supine position; (**g**) HR, (**h**) mean HR, (**i**) LFnu, (**j**) HFnu, (**k**) LF/HF, and (**l**) RR in the sitting position.

Figure 4 shows the results of the Bland–Altman analysis of the HR, LFnu, HFnu, LF/HF, and RR extracted using the proposed system and those measured using the commercial instruments. The 95% confidence intervals of the indices obtained in the supine position are as follows: HR: −5.26–5.54 bpm; mean HR: −0.47–0.56 bpm; LFnu: −0.24–0.12 a.u.; HFnu: −0.12–0.24 a.u.; LF/HF: −1.70–1.05 a.u.; RR: −2.58–2.90 breaths/min. The 95% confidence intervals of the indices obtained in the sitting position are as follows: HR: −8.91–8.46 bpm; mean HR: −1.83–2.46 bpm; LFnu: −0.25–0.18 a.u.; HFnu: −0.18–0.25 a.u.; LF/HF: −1.34–1.14 a.u.; RR: −2.43–3.50 breaths/min. In the Bland–Altman plots, the constant errors between extracted signals measured by the proposed system and those measured by the commercial instruments were not confirmed under all conditions in the Bland–Altman analysis. Proportional errors were seen for HR, LF/HF, and RR in the supine position and for mean HR, LF/HF, and RR in the sitting position. However, both the slope of the regression lines and the correlation coefficients between the averages and errors of HR, mean HR, LF/HF, and RR are small. The proportional errors can thus be small (supine position: HR, *r* = −0.12, *p* < 0.001, slope of the regression line *a* = −0.03; LF/HF, *r* = −0.30, *p* < 0.001, slope of the regression line *a* = −0.12; RR, *r* = −0.32, *p* < 0.001, slope of the regression line *a* = −0.23; sitting position: mean HR, *r* = −0.17, *p* < 0.001, slope of the regression line *a* = −0.02; LF/HF, *r* = 0.22, *p* < 0.01, slope of the regression line *a* = 0.15; RR, *r* = −0.18, *p* < 0.05, slope of the regression line a = 0.16).

### Changes in extracted vital signs by affect

**Figure 5:**
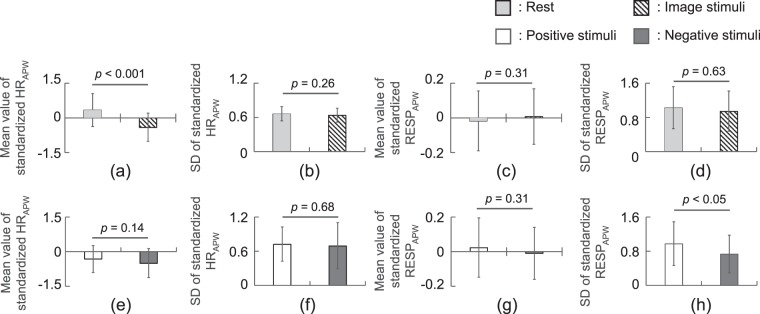
Change in vital signs between the periods of rest and periods in which image stimuli were displayed: (**a**) mean value of standardized HR_APW_, (**b**) SD of standardized HR_APW_, (**c**) mean value of standardized RESP_APW_, and (**d**) SD of standardized RESP_APW_; Comparison of vital signs measured during the display of positive and negative image stimuli: (**e**) mean value of standardized HR_APW_, (**f**) SD of standardized HR_APW_, (**g**) mean value of standardized RESP_APW_, and (h) SD of standardized RESP_APW_.

Figure 5 compares the extracted signals (mean value and SD of HR_APW_ and RESP_APW_) for the stimulus period and those for the rest state; similarly, it compares the extracted signals for the positive stimulus period and those for the negative stimulus period. The mean value of HR_APW_ during image stimulation significantly decreased compared with that at rest (*p* < 0.001). The SD of RESP_APW_ during negative image stimulation significantly decreased compared with that during positive image stimulation (*p* < 0.05).

Table 2 shows the PCA results of the self-reported VAS scores of affect. The number of extracted principal component scores was three, under the condition that the cumulative contribution ratio was greater than 80%. The first principal component (PC1) is positively correlated with aroused, bored, displeased, and irritated, and it is negatively correlated with excited, pleased, relaxed, and sleepy. The second principal component (PC2) is positively correlated with sleepy, bored, displeased, and irritated, and negatively correlated with aroused, excited, pleased, and relaxed. The third principal component (PC3) is positively correlated with aroused, excited, pleased, relaxed, and bored, and negatively correlated with sleepy, displeased, and irritated.

**Table 2:** PCA.

Index	PC1	PC2	PC3
Aroused	0.30	−0.47	0.39
Excited	−0.39	−0.05	0.24
Pleased	−0.42	−0.02	0.17
Relaxed	−0.41	−0.05	0.20
Sleepy	−0.30	0.47	−0.44
Bored	0.20	0.70	0.68
Displeased	0.43	0.03	−0.14
Irritated	0.32	0.24	−0.23
Cumulative contribution ratio [%]	62.3	75.2	84.1

Table 3 shows the relationships between extracted signals and the self-reported affect VAS scores and corresponding images. The calculated values of HR_APW_ and RESP_APW_ are correlated to the following several self-reported affect values: mean value of HR_APW_ and relaxed (*r* = 0.26, *p* < 0.05), mean value of RESP_APW_ and excited (*r* = 0.23, *p* < 0.05), SD of RESP_APW_ and excited (*r* = 0.28, *p* < 0.01), SD of RESP_APW_ and pleased (*r* = 0.21, *p* < 0.05), SD of RESP_APW_ and relaxed (*r* = 0.22, *p* < 0.05), and SD of RESP_APW_ and displeased (*r* = −0.23, *p* < 0.05)). A significant correlation between PC1 and the SD of RESP_APW_ was found (*r* = −0.21, *p* < 0.05). A significant correlation between PC2 and the SD of HR_APW_ was also found (*r* = −0.23, *p* < 0.05). The SD of RESP_APW_ was significantly correlated with the total degree of pleasedness/displeasedness, and the SD of HR_APW_ was significantly correlated with the total degree of aroused/sleepiness. There was no significant correlation between PC3 and the vital signs.

**Table 3:** Correlation coefficient results.

Index	HR_APW_		RESP_APW_	
	Mean	SD	Mean	SD
Aroused	−0.17	0.17	0.10	−0.13
Excited	0.14	0.03	0.23*	0.28**
Pleased	0.18	0.02	0.02	0.21*
Relaxed	0.26*	−0.01	0.05	0.22*
Sleepy	0.17	−0.12	−0.01	0.02
Bored	−0.09	−0.07	0.01	−0.02
Displeased	−0.19	0.01	−0.11	−0.23*
Irritated	0.02	−0.17	−0.06	0.16
PC1	−0.20	0.003	−0.07	−0.21*
PC2	0.06	−0.23*	−0.08	−0.02
PC3	−0.06	0.14	0.17	0.16

*p < 0.05, **p < 0.01.

## Discussion

The authors proposed an algorithm to achieve unconstrained, simultaneous monitoring of vital signs (i.e., heart rate, respiratory rate, and HRV indices) using APWs measured by APW sensors. Two experiments were conducted to verify the effectiveness of the proposed algorithm.

In the first experiment, we compared the vital signs obtained by the proposed system and those measured by commercial instruments. The results showed significantly high correlation between the systems (see Table 1); however, we also found that the correlations were lower for seated participants than for supine participants. Although the reason for this remains unclear, we believe that it occurred because participants pushed the APW sensors downward with their backs when sitting down, and this point should be improved in future work. Nonetheless, the high correlations indicate that the proposed system can accurately extract vital signs from APWs, particularly for patients in the supine position. The proposed system provides a distinctive advantage over previous systems^9–12^ in that it embeds APW sensors inside the 3D-NET^19,21^, a design that prevents decubitus in bedridden patients. Therefore, the proposed system can be used with a wide range of users, from healthy to bedridden. In addition, the high sampling rate (1 kHz) of the proposed system provides sufficiently high reliability for extracting HRV indices.

We performed image stimulation experiments and compared the extracted vital signs with the affect subjectively rated by the participants. To this end, we performed principal component analysis on the rated affect indices (see Table 2). Using the affect list shown in Table 2, an interpretation of the extracted principal components is provided below. PC1 explains pleased/displeased (main positive direction: displeased and irritated; main negative direction: excited, pleased, and relaxed). PC2 and PC3 explain aroused/sleepiness. The positive direction of PC2 is primarily a pleased affect, while that of PC3 is primarily a displeased affect; PC2 and PC3 are components that reflect aroused. The principal components extracted in this experiment were thus similar to those of Russell’s circumplex model of affect^31^, which expressed human affect in a two-dimensional circumplex model of arousal and pleasedness. This result indicates that the affect responses obtained in this experiment were reasonable.

We also confirmed that vital signs changed when image stimuli were displayed (see Fig. 5). The heart rate decreased when image stimuli were displayed, which is a prototypical finding in psychophysiological investigations^34^. In particular, the heart rate significantly decreased when negative images were displayed. In previous studies, three types of images (positive, negative, and neutral) from the IAPS were presented to participants, and changes in the vital signs were confirmed. Drops in heart rates are attributed to the participant paying greater attention to a negative image than to a positive image^35,36^. The results of our study are consistent with those of previous studies. In addition, our study newly finds that the respiration extracted using the proposed system reflected the pleased/displeased affect of the participants. These results indicate that the proposed system can be applied to measure affect in humans.

In conclusion, this study proposed a novel unconstrained monitoring system that can measure heart rate, respiratory rate, and evaluate autonomic nervous activity based on HRV in supine and seated patients using a microphone-type APW sensor. The extracted vital signs changed depending on the affect of the participant. The proposed system can be installed in a chair or bed for health condition management during daily activities, and it can log fatigue and feelings. In future work, we will consider the implementation of long-term monitoring using the proposed system and incorporation into actual home health care.
